# Detailed analysis of phenotypes and genotypes in megalencephaly-capillary malformation-polymicrogyria syndrome caused by somatic mosaicism of *PIK3CA* mutations

**DOI:** 10.1186/s13023-020-01480-y

**Published:** 2020-08-10

**Authors:** Hyun Jin Park, Chang Ho Shin, Won Joon Yoo, Tae-Joon Cho, Man Jin Kim, Moon-Woo Seong, Sung Sup Park, Jeong Ho Lee, Nam Suk Sim, Jung Min Ko

**Affiliations:** 1 Department of Pediatrics, Seoul National University Children’s Hospital, Seoul National University College of Medicine, Jongnogu Daehakro 101, Seoul, 03080 Republic of Korea; 2 Division of Pediatric Orthopaedics, Department of Orthopaedic Surgery, Seoul National University Children’s Hospital, Seoul National University College of Medicine, Seoul, Republic of Korea; 3 Department of Laboratory Medicine, Seoul National University Hospital, Seoul National University College of Medicine, Seoul, Republic of Korea; 4 Rare Disease Center, Seoul National University Hospital, Seoul, Republic of Korea; 5 Graduate School of Medical Science and Engineering, KAIST, Daejeon, Republic of Korea

**Keywords:** PIK3CA, Somatic overgrowth, Megalencephaly, Asymmetry, Cutaneous vascular malformation

## Abstract

**Background:**

Megalencephaly-capillary malformation-polymicrogyria syndrome (MCAP) belongs to a group of conditions called the *PIK3CA*-related overgrowth spectrum (PROS). The varying phenotypes and low frequencies of each somatic mosaic variant make confirmative diagnosis difficult. We present 12 patients who were diagnosed clinically and genetically with MCAP. Genomic DNA was extracted mainly from the skin of affected lesions, also from peripheral blood leukocytes and buccal epithelial cells, and target panel sequencing using high-depth next-generation sequencing technology was performed.

**Results:**

Macrocephaly was present in 11/12 patients (92%). All patients had normal body asymmetry. Cutaneous vascular malformation was found in 10/12 patients (83%). Megalencephaly or hemimegalencephaly was noted in all 11 patients who underwent brain magnetic resonance imaging. Arnold–Chiari type I malformation was also seen in 10 patients. Every patient was identified as having pathogenic or likely pathogenic variants of the *PIK3CA* gene. The variant allele frequency (VAF) ranged from 6.3 to 35.3%, however, there was no direct correlation between VAF and the severity of associated anomalies. c.2740G > A (p.Gly914Arg) was most commonly found, in four patients (33%). No malignancies developed during follow-up periods.

**Conclusions:**

This is the first and largest cohort of molecularly diagnosed patients with MCAP in Korea. Targeted therapy with a PI3K-specific inhibitor, alpelisib, has shown successful outcomes in patients with PROS in a pilot clinical study, so early diagnosis for genetic counseling and timely introduction of emerging treatments might be achieved in the future through optimal genetic testing.

## Introduction

Megalencephaly-capillary malformation-polymicrogyria syndrome (MCAP; MIM #602501) is a rare genetic disorder characterized by megalencephaly, cutaneous capillary malformation, and somatic overgrowth. It is known that MCAP is caused by somatic mosaicism of the *PIK3CA* gene and is one type of disorder in the *PIK3CA*-related overgrowth spectrum (PROS). Several segmental overgrowth syndromes, including congenital lipomatous overgrowth, vascular malformations, epidermal nevi, scoliosis/skeletal and spinal syndrome (CLOVES, MIM #612918), fibroadipose hyperplasia or overgrowth (FAO), and hemihyperplasia multiple lipomatosis (HHML), have also turned out to result from activating variants in PIK3CA and are categorized as PROS. The phenotypes of patients with MCAP are more severe than for other types of PROS and show pleiotropy [1–3].

The phosphatidylinositol 3-kinase (PI3K)/protein kinase B (AKT)/mammalian target of the rapamycin (mTOR) pathway plays a crucial role in regulating cell growth and survival. Dysregulation and overactivation of this pathway can lead to tumorigenesis. Therefore, it has been one of the most attractive targets for anticancer therapy. *PIK3CA* is a gene encoding the p110α protein that is a catalytic subunit of class IA PI3K. Postzygotic variants of *PIK3CA* are associated with diverse cancers, especially breast cancers [4]. With advances in DNA sequencing technology, somatic mosaic variants of *PIK3CA* have also been identified as causative in several overgrowth syndromes.

The causative gene might have been revealed, but MCAP is still difficult to diagnose both clinically and genetically because the clinical manifestations and severity of each set of symptoms vary from patient to patient, and somatic variants can only be found through the proper method using adequate samples. To date, the only effective treatment for PROS is surgical resection in cases having an organ or tissue dysfunction caused by severe overgrowth. However, a newly developed target agent, a PI3K-specific inhibitor (alpelisib; Novartis AG, Basel, Switzerland), has shown successful outcomes in animal models of PROS and patients with CLOVES [5]. A phase I clinical trial of this drug for patients with PROS is now in preparation [6]. Although the cancer risk is uncertain for such cases, regular follow-up and tumor surveillance are recommended based on several cases of tumors [7–9]. Hence, precise clinical and molecular diagnostic methods for PROS have grown to prominence given the emerging treatment and tumor surveillance options.

Here, we report on the clinical and molecular genetic characteristics of 12 Korean patients confirmed as having MCAP. Using high-depth next-generation sequencing (NGS) techniques and focused analysis for genes related to PI3K/AKT/mTOR pathway, we could detect low allele frequencies of somatic mosaic variants successfully from the patients’ blood and affected tissues.

## Methods

### Patients

From 1998 to 2019, we reviewed 12 patients from 12 families who satisfied the clinical MCAP criteria and were confirmed genetically to have *PIK3CA* pathogenic variants. We employed the latest diagnostic criteria proposed by Martinez-Glez et al. [10]. For the diagnosis, a patient should exhibit three major and two minor criteria. Major criteria comprise macrocephaly, capillary malformation, overgrowth/asymmetry and neuroimaging alterations; ventriculomegaly, cavum septum pellucidum, or cavum vergae, cerebellar tonsillar herniation (Arnold–Chiari type I malformation); and cerebral and/or cerebellar asymmetry. Minor criteria comprise developmental delay, midline facial capillary malformations, neonatal hypotonia, digital anomalies including syndactyly and polydactyly, frontal bossing, connective tissue abnormality, and hydrocephalus.

The initial chief complaints at presentation, past medical history, and results of previously performed genetic tests were collected retrospectively. The patients were evaluated with various imaging studies including brain magnetic resonance imaging (MRI; *n* = 11), teleradiograms of lower extremities (*n* = 10) and abdominal sonography (*n* = 9). They were checked regularly for developmental status and somatic growth (height, weight, and head circumference) for a mean duration of 6.0 years (range 0–20.9).

### Molecular genetic analysis

We performed target panel sequencing using high-depth NGS technology in all 12 patients. Genomic DNA was extracted from affected skin lesions (*n* = 10), buccal epithelial cells (*n* = 2), and peripheral leukocytes (*n* = 5). Four patients (P1–4), underwent sequencing through both peripheral blood and skin specimens. P8 was tested using both skin lesions and buccal epithelial cells.

The amount of double-stranded DNA was quantified using a Qubit Fluorometer 4.0 (Invitrogen Life Technologies, Waltham, MA, USA). At least 500 ng of genomic DNA is required for deep gene panel sequencing. After hybrid capture, the quantity of captured library was determined using the Qubit system. For hybrid capture, sheared genomic DNA 150–300 base pairs (bp) in length was quantified using an Agilent 2100 Bioanalyzer (Agilent Technologies, Santa Clara, CA, USA). The library of genomic DNA 200–500 bp length was quantified similarly.

For genetic analysis, we designed a targeted gene hybrid capture sequencing panel with Celemics Inc. (Celemics, Seoul, S. Korea). The panel included 11 mTOR pathway genes: *AKT1*, *AKT3*, *DEPDC5*, *MTOR*, *NPRL2*, *NPRL3*, *PIK3CA*, *PIK3R2*, *PTEN*, *TSC1*, and *TSC2*. The performance of the panel used for targeted sequencing was explored using TarSeqQC (https://www.bioconductor.org/packages/release/bioc/html/TarSeqQC.html) [11]. Library preparation was performed according to the manufacturer’s protocol. Briefly, all genomic DNA sequences were fragmented to ~ 200 bp using a Focused-ultrasonicator S2 (Covaris Inc., Woburn, MA, USA). After end-repair, A-tailing, and adaptor ligation processes, we prepared two independent targeted gene libraries from each sample using the biotin–streptavidin complex method. Each replicated library from hybrid capture was sequenced on a Miseq Dx sequencer (Illumina, Inc., San Diego, CA, USA) by SoVarGen (Daejeon, S. Korea). To increase the somatic variant detection rate, the mean depth in sequencing was set up as high as possible, at least 500× and the coverage target region over 100× was 100%.

We analyzed the sequencing data as described [12]. We aligned raw sequences from Fastq files to the hg19/GRCh37 assembly of the human genome reference sequence using BWA-MEM (http://bio-bwa.sourceforge.net). After recommended preprocessing, indel realignment, and base recalibration, we utilized RePlow (https://sourceforge.net/projects/replow/), a variant caller designed to detect low-level somatic variants from replicated sample data [13]. We then applied in-house filtering criteria: 1) excluding registered common variants in a public database (common dbSNP147; https://genome.ucsc.edu/cgi-bin/hgTrackUi?db=hg38&g=snp147Common); 2) excluding variants with a putative low snpEFF impact score (http://snpeff.sourceforge.net/SnpEff.html#intro); 3) excluding variants with a PolyPhen & SIFT ≠ Damaging, phastCons score < 0.9; and 4) excluding variants with an allele frequency > 0.1% in the ExAC and gnomAD databases (both from https://gnomad.broadinstitute.org) for minor allele frequencies of general and East Asian populations [14]. To detect germline variants, we used GATK HaplotypeCaller (https://gatk.broadinstitute.org/hc/en-us) with hard filters. For our analysis of germline variants, we evaluated variants according to the American College of Medical Genetics and Genomics guidelines [15]. According to these, which are particularly useful for interpretation of germline sequence variants, missense variants cannot be assumed to be pathogenic if there is no evidence from functional studies and no confirmation of de novo variants.

## Results

### Clinical characteristics

**Fig. 1:**
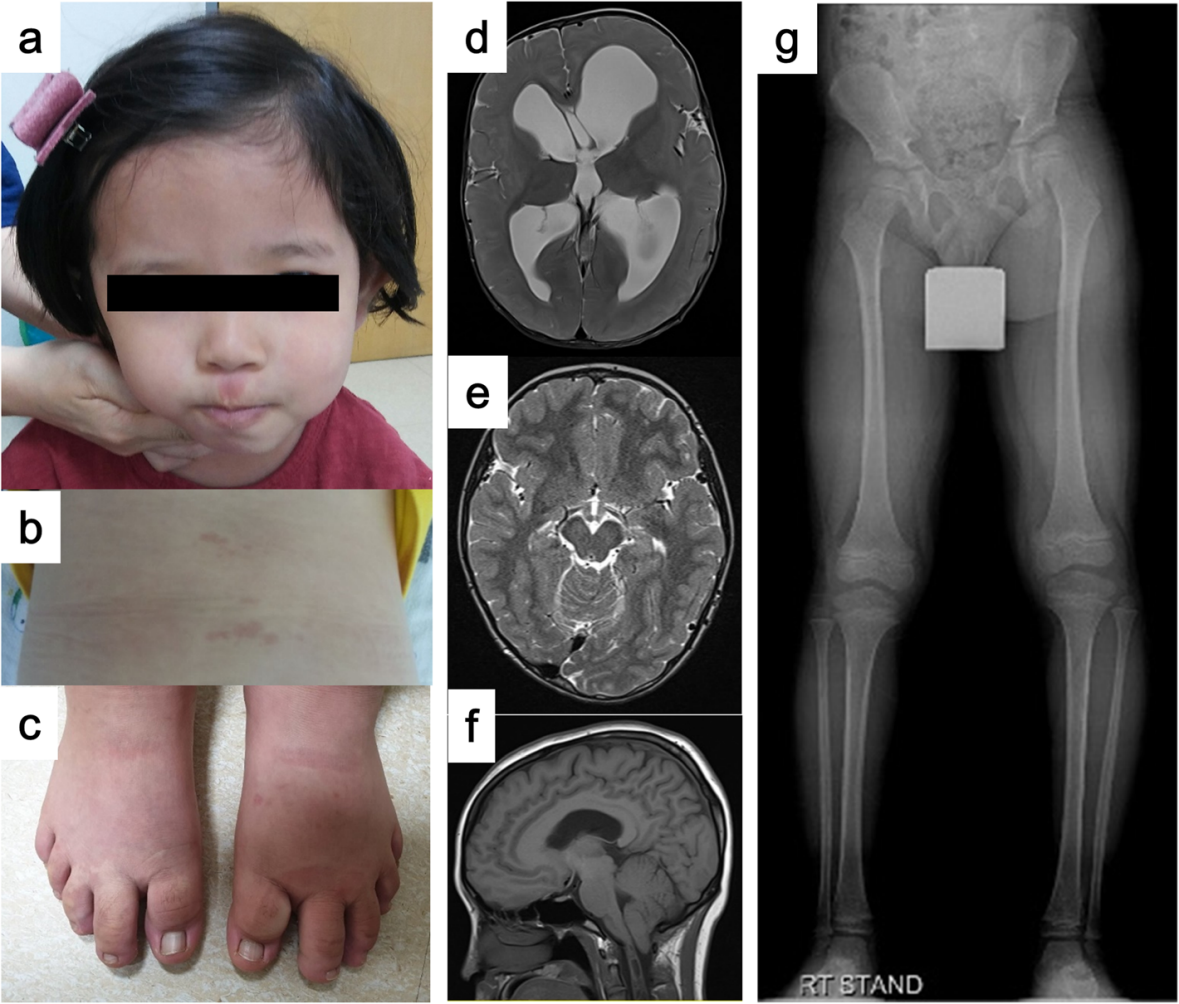
Images of the patients showing various clinical manifestations. **a** Frontal bossing, facial asymmetry, and cutaneous capillary malformation, hemangioma-like lesions on the philtrum. **b** Cutaneous capillary malformation on the back. **c** Hypertrophy of bilateral first and second toes. **d**, **e** Left-sided hemimegalencephaly. **f** Arnold–Chiari type I malformation. **g** Leg length discrepancy is shown in a simple radiograph

Table 1 summarizes the clinical characteristics of the patients. Their typical MCAP phenotypes are shown in Fig. 1. There were seven female and five male patients. The median age at molecular confirmation was 7.6-year-old (range 3.0–21.0). The mean follow-up duration from the initial presentation of symptoms or signs was 6.0 years (range 1–20.9). All patients were de novo cases and there was no familial history of an overgrowth syndrome.

**Table 1:** Summary of clinical manifestations and identified *PIK3CA* mutations in our patients

	P1	P2	P3	P4	P5	P6	P7	P8	P9	P10	P11	P12	Total	Ref [10, 16-19]
Sex	M	M	F	F	M	F	M	F	M	F	F	F	F 7/12 (58%)	15–41%
Age^a^	3 y 10 m	6 y 4 m	3 y 6 m	4 y 4 m	11 y 7 m	21 y	6 y 2 m	3 y	10 y 2 m	12 y 11 m	3 y 5 m	4 y 3 m		
FU duration	3 y 5 m	5 y 4 m	3 y 3 m	1 y 2 m	9 y 4 m	20 y 5 m	5 y 1 m	1 y	0 m	12 y 10 m	3 y 3 m	3 y 9 m		
Mutation^b^	+	+	+	+	+	+	+	+	+	+	+	+		
Specimen	Skin	Skin	PB, Skin	Skin	PB	skin	skin	Skin, Buccal	skin	skin	skin	Buccal		
Identified variant	c.1359_1361 delAGA (p.Glu453del)	c.263G > A (p.Arg88Gln)	c.3139C > T (p.His1047Tyr)	c.1359_1361 delAGA (p.Glu453del)	c.2740G > A (p.Gly914Arg)	c.1133G > A (p.Cys378Tyr)	c.2740G > A (p.Gly914Arg)	c.1635G > T (p.Glu545Asp)	c.2740G > A (p.Gly914Arg)	c.241G > A (p.Glu81Lys)	c.2740G > A (p.Gly914Arg)	c.3193C > T (p.His1065Tyr)		
VAF	20.1	33.0	35.0 (Skin) 35.3 (PB)	23.0	17.9	31.0	27.6	6.3 (Skin) 13.5 (Buccal)	10.0	34.9	18.7	17.7		
Perinatal history														
GA (weeks)	32 + 3^c^	40 + 4	34 + 0^c^	38 + 0	NA	38 + 0	37 + 0	39 + 5	38 + 0	38 + 0	39 + 0	38 + 0	Pre-2/11 (18%)	23–33%
Birth weight (percentile of normal)	2.42 93	3.94 70	2.85 95	3.8 93		3.6 86	4.18 100^d^	3.4 53	4.14 98	4.7 100	3.2 47	3.5 82	LGA^g^ 6/11 (55%)	44–100%
Physical measurement (SD) at the last outpatient clinic visit														
Height	−1.57	−0.21	−2.57	+ 0.65	NA	−0.86	+ 1.71	−1.68	−0.62	−0.90	−0.76	−0.62		
Weight	+ 1.16	+ 1.69	−1.94	+ 0.83		+ 1.63	+ 2.69	+ 0.15	+ 0.27	+ 1.20	−0.89	+ 0.11		
OFC	+ 4.23	+ 2.99	+ 2.62	+ 2.62	+ 7.74	+ 5.57	+ 3.69	+ 3.03	+ 5.38	+ 3.27	+ 0.74	+ 3.16	11/12 (92%)	85–100%
Segmental overgrowth	+ LLD (Lt > Rt)	+ LLD (Lt > Rt)	+ Generalized (Lt > Rt)	+ LLD (Lt > Rt)	+ ^d^ Hands/feet	+ LLD (Lt > Rt)	+ LLD (Lt > Rt)	+ Face (Rt > Lt) LLD (Lt > Rt)	+ LLD (Rt > Lt)	+ LLD (Rt > Lt)	+ LLD (Rt > Lt)	+ LLD (Lt > Rt)	12/12 (100%) LLD 11/12 (92%)	69–100% 31–88%
Brain MRI Findings														
(H)MEG	+	+	+, Lt	+, Rt	+	+	+	+	NA	+	+, Rt	+	11/11 (100%)	55–83%
PMG	–	+	–	–	±	–	–	–		–	–	–	2/11(18%)	17–79%
A-C type I	+	+	+	+	–	+	+	+		+	+	+	10/11 (91%)	0–90%
VP shunt	+	–	+	–	–	+	–	–	–	–	–	–	3/12 (25%)	25–52%
DD or MR	–	±^e^	+	–	+	+	+	–	–	–	–	–	5/12 (42%)	60–90%
Seizure	–	+	–	–	+	–	–	–	–	–	–	–	2/12 (18%)	16–24%
Cut VMs	+ Face, back	+ Back	–	+ Face	–	+ Face (philtrum)	+ Face (forehead)	+ Face (philtrum)	+ Face, back (philtrum)	+ Whole body	+ Face, trunk, leg, feet	+ Face (forehead)	10/12 (83%) Midline 6/12 (50%)	82–100% 58–86%
Digital anomaly	S	S	–	–	–	S	–	S	P	M^f^	M^f^	–	S + P 5/12 (42%)	42–67%
Other anomaly	–	Meningo-myelocele	ASD/Bilateral SVC/Tracheal stenosis	–	–	–	Nephro-megaly	–	–	–	–	–		

Key: Digital anomaly (*S* Syndactyly, *P* Polydactyly, *M* Macrodactyly), *M* Male, *F* Female, *y/m* Year/month, *FU* Follow-up, *PB* Peripheral blood, *buccal* Buccal epithelial cells, *VAF* Variant allele frequency, *GA* Gestational age, *Pre* Preterm, *LGA* Large for gestational age, *SD* Standard deviation, *OFC* Occipitofrontal circumstance, *NA* Not available (P5 did not have the height or weight recorded; P9 did not undergo brain MRI because of parental disapproval), *LLD* Leg length difference, *DD* Developmental delay, *MR* Mental retardation, *(H)MEG* (Hemi-) or megalencephaly, *PMG* Polymicrogyria, *Lt* Left, *Rt* Right, *A-C type I* Arnold–Chiari type I malformation, *VP* Ventriculoperitoneal, *Cut. VMs* Cutaneous vascular malformation, *ASD* Atrial septal defect, *SVC* Superior vena cava

^a^ Age at molecular diagnosis

^b^ All patients were identified as having *PIK3CA* mosaic mutations

^c^ P1 and P3 were born preterm due to preterm premature rupture of membranes

^d^ Maternal gestational diabetes mellitus

^e^ Borderline intellectual functioning (FSIQ = 86)

^f^ No definite syndactyly or polydactyly but remarkably large toes

^g^ Large for gestational age is defined as a birth weight greater than 90th percentile of normal

Ten patients (83%) were born full-term and two (17%) were born preterm because of preterm premature rupture of membranes. Four patients (33%) had a large birth weight for gestational age (>90th percentile) and the mother of one of them (P7) had gestational diabetes mellitus. The remaining patients (67%) had appropriate weights for gestational age. Only one patient (P3) had an abnormality shown by fetal ultrasonography: ventriculomegaly, asymmetric brain parenchyma, a single umbilical artery and nuchal cord. One patient (P6) was conceived using assisted reproductive technology.

On physical examinations, head circumference was larger than + 2.0 standard deviations (SDs) of the normal in 11 patients (92%). The heights and weights of patients were less than + 2.0 SD from the normal range except for the weight of P7. All the patients had segmental overgrowth; P5 had hypertrophy in both hands and feet and P8 showed facial asymmetry. The other patients (83%) had leg length discrepancies with a range of 1.0–3.0 cm on simple radiographs. P10 underwent percutaneous epiphysiodesis surgery for the progressive leg length discrepancy.

Brain MRI was performed in 11 patients, and megalencephaly (*n* = 8) or hemimegalencephaly (*n* = 3) was identified in all. Arnold–Chiari type I malformation coexisted in 10 of these patients (91%). Three patients (3 of 12, 25%) underwent a ventriculoperitoneal (VP) shunt operation for hydrocephalus, and P6 needed a second operation for decompression of the foramen magnum. Four patients (33%) showed apparent developmental delay and one, P2, had borderline poor intellectual functioning, scoring 86 on a full-scale intelligence quotient evaluation. Cutaneous vascular malformation was noted in 10 patients (83%) and this lesion was most frequently shown on the face (75%). These skin lesions improved with age in most cases. Digital anomalies including syndactyly (*n* = 4), polydactyly (*n* = 1), and macrodactyly (*n* = 2) were combined in seven of the patients (58%), and P6 and P9 required surgical correction. In addition, meningomyelocele was corrected surgically in P2, and an atrial septal defect, bilateral superior vena cava, and severe tracheal stenosis requiring tracheostomy were also seen in P3. Nephromegaly ipsilateral to hemihypertrophy was observed in P7. No case had any malignancy developing during the follow-up periods under regular surveillance with abdominal ultrasonography.

The chief complaints at the initial hospital visit were body asymmetry in nine patients (75%), macrocephaly in two (17%), and developmental delay in one (8%). Before the molecular confirmation of MCAP, eight patients (67%) had been subjected to several genetic evaluations: the mean number per patient was 2.8 (range 1–4). Methylation studies for Beckwith–Wiedemann syndrome was most frequently performed, in five of the eight patients. Conventional karyotyping, chromosomal microarray, and whole-exome sequencing (WES) using peripheral blood were done for four, two, and two patients, respectively. Urinary glycosaminoglycan analysis for the presence of mucopolysaccharidosis was done in two patients, and screening tests for other metabolic disorders were done in one (P5). Sanger sequencing of *NSD1*, *PTEN*, and *FGFR3* using peripheral blood was performed for each patient, and peripheral blood samples of two also underwent Sanger sequencing of *PIK3CA*. All these tests failed to reveal the genetic etiology of the patients’ symptoms and signs.

### PIK3CA genotypes

**Fig. 2:**
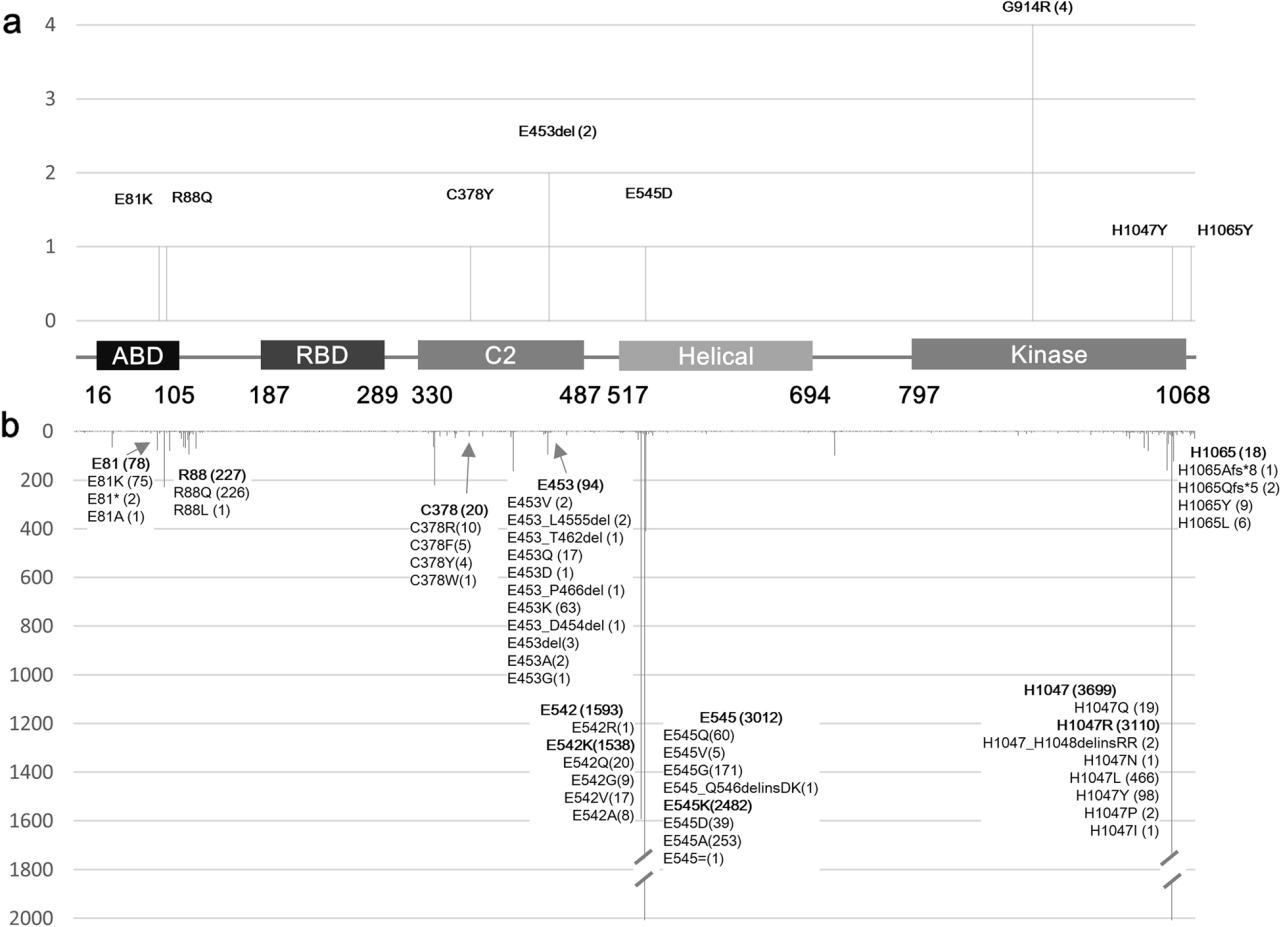
Distribution of *PIK3CA* variants identified in this study and cancers. **a** The graph shows the variants found in our 12 patients diagnosed with MCAP. Eight variants were identified and p.Gly914Arg was the most frequent. **b** The number of reported variants reported in cancers is arranged, using data from the Catalogue Of Somatic Mutations In Cancer (COSMIC, https://cancer.sanger.ac.uk/cosmic, released September 2019). Amino acid locations that were found in this study or were “hotspots” are marked separately. Common variants (p.Glu542Lys, p.Glu545Lys, and p.His1047Arg) are in bold

Causative somatic variants found in *PIK3CA* are listed in Table 1 and shown in Fig. 2. All detected *PIK3CA* variants have been reported previously in patients with PROS or various cancers, and could be classified as pathogenic or likely pathogenic variants in online databases including ClinVar (http://www.ncbi.nlm.nih.gov/clinvar) and COSMIC (Catalogue Of Somatic Mutations In Cancer, http://cancer.sanger.ac.uk/cosmic). Eight causative variants were identified from these patients. The most commonly found variant was p.Gly914Arg in four (33%), followed by p.Glu453del in two (17%). The other six variants (p.Glu81Lys, p.Arg88Gln, p.Cys378Tyr, p.Glu545Asp, p.His1047Tyr, and p.His1065Tyr) were found each in one patient. One variant, p.Glu453del, was an in-frame deletion and the others were missense variants. The overall mean variant allele frequency (VAF) for identified pathogenic variants was 22.2% (range 6.3–35.3%).

Origins of tested DNA were as follows: skin affected by cutaneous vascular malformation (*n* = 10), peripheral blood (*n* = 5), and buccal epithelial cells (*n* = 2). Genetic etiology was revealed in all patients tested using skin or buccal specimens. In P8, tested with two tissue samples, VAF was higher in the buccal sample (6.3% for skin vs 13.5% for buccal cells). We also could detect mosaic pathogenic variants from peripheral blood DNA in two patients (P3 and P5). In P3, mosaicism of *PIK3CA* was not detected through WES but identified through high-depth target panel sequencing. Simultaneous analyses of DNA from skin and peripheral blood was performed for P3, and the VAFs were 35.0 and 35.3%, respectively. P1 and P2 had been evaluated previously for the *PIK3CA* gene in peripheral blood through Sanger sequencing. No pathogenic variant was detected in the blood but it was in the skin. For P7, the pathogenic variant was not found through trio-WES with blood but detected through deep sequencing with a skin specimen.

## Discussion

Overgrowth is a common characteristic shown in diverse congenital syndromes. It is not a single symptom but a spectrum of diseases ranging from mild asymmetric macrosomia to megalencephaly associated with an intellectual disability or tumor-prone conditions. Because the actual manifestations differ widely between patients depending on the affected organ and degree of impact, it is hard to recognize these manifestations as a solitary category. However, there has been an effort to classify these syndromes based on similar overgrowth symptoms, leading to an effort to find the causative gene for each disease group. Through conventional genetic evaluations, germline variants were identified first, such as *NF1* for neurofibromatosis and *NSD1* for Sotos syndrome [20]. Then, mosaicisms were recognized as other causative mechanisms of overgrowth syndromes, and several of the genes involved, including *AKT1*, *PTEN*, and *PIK3CA*, have been identified [21–23]. Some reports found that the rate of detection of somatic mosaicism in overgrowth syndromes improved using high-sensitivity sequencing with specimens of the affected tissues rather than peripheral blood [23–26]. Here, we selected a group of patients who met strict phenotypic diagnostic criteria of MCAP and conducted high-depth target gene sequencing, mostly with affected tissues. As a consequence, we found pathogenic or likely pathogenic variants of *PIK3CA* in all the patients. This result emphasizes the importance of phenotype analysis and clinical impression.

Comparing the frequency of each typical phenotype in this study with those found in previous studies [10, 16–19], our patients mostly showed similar frequencies in phenotypes, especially the major criteria of macrocephaly (92% vs 85–100%), capillary malformation (83% vs 82–100%), overgrowth/asymmetry (100% vs 69–100%), and neuroimaging alterations (100% vs 55–90%). Other minor features had relatively wide frequency ranges in previous studies, such as developmental delay (60–90%), midline facial capillary malformation (58–86%), neonatal hypotonia (17–92%), syndactyly or polydactyly (42–67%), frontal bossing (6–42%), connective tissue abnormalities (33–100%), and hydrocephalus (25–52%). One possible reason could be that the specialties and interests of each investigating group differed. Besides the primary indications, macrocephaly and capillary malformation, combined symptoms or recognition of signs could differ depending on each physician’s point of view. In our case, most patients (75%) were referred to our clinic for treating hemihypertrophy from orthopedic surgeons, and there were only two cases of macrocephaly and one of developmental delay. In addition, because our main indication for evaluating MCAP was macrocephaly, the frequency of that feature was higher than in previous reports. Hypotonia is usually prominent in infants and young children, and improves with age. Therefore, hypotonia might have been under-recognized because all of our patients were older than 3 years of age at their first visit.

There have been attempts to distinguish phenotypes and common variants between MCAP and non-MCAP syndromes within a single disease spectrum—PROS—to identify genotype–phenotype correlations [27, 28]. MCAP is mostly accompanied by megalencephaly, but other somatic overgrowth symptoms, such as lipoma and severe vascular or lymphatic malformations, are relatively rare or mild. This scenario differs from non-MCAP PROS, as seen in CLOVES and FAO. The results of our study are consistent with those of the previous studies [27, 28]. The frequency of somatic overgrowth except macrocephaly was high, but this feature was not as severe. Furthermore, there was no case combined with lipoma or lymphatic malformations and only one patient (P10) required surgery for a progressive leg length discrepancy during the follow-up. Most of the cutaneous vascular lesions have improved.

As to the genotypes found in here, there was no novel variant. All the variants except p.His1065Tyr have been reported in cases of PROS previously. This variant was only found in nine cases of cancers and is the first one found in a patient with PROS. We could not identify a mutational hotspot in *PIK3CA* for causing MCAP that could distinguish it from non-MCAP PROS, and that is similar to the results of previous studies [24, 27–29]. In addition, we matched the oncogenic activity of each variant according to Dogruluk et al. [30] and except for one variant, p.His1047Tyr, with intermediate activity, the others have unknown activities for tumorigenesis. The patient (P3) with p.His1047Tyr was the most severe case of our study cohort, and was the only one who showed abnormal fetal growth and multiple congenital anomalies combined with severe mental retardation.

There seemed to be no direct correlation between VAF and the severity of phenotypes, but some limitations prevented us from evaluating this precisely. First, even if the type of specimen was identical, the degree of overgrowth differed among patients. Moreover, it would be ideal to collect a specimen from the most severely affected lesion, but the accessibility and safety of sampling cannot be ignored. A recent study compared VAFs between different samples taken by autopsy of a severe case of PROS [31]. However, there was no definite correlation between the phenotypic severity and VAF findings, even from the same type of tissue such as skin. In other studies of larger cohorts, researchers could also not find any correlation between VAF and phenotypes [25, 27, 28].

In our study, two of five cases tested using peripheral blood samples showed the presence of mosaicism. Both of these patients showed developmental delay, and one of them (P3) was the most severe case in our cohort, having multi-organ involvement. Although two cases are clearly insufficient for any firm conclusion, this observation supports the idea that PROS-related *PIK3CA* variants detectable in blood may occur at an earlier embryonic stage and affect more organ systems. In addition, these variants are mostly related to MCAP [8, 25, 28]. On the other hand, all two cases tested using buccal epithelial cells were confirmed *PIK3CA* mosaicism. In P8, who was tested from skin and buccal samples, the VAF in buccal cells was higher than in skin. This result is in agreement with previous reports that the VAF and detection rate using saliva or buccal specimen were higher than when using blood [23, 24, 27, 28]. One reason might be the different embryonic lineages between blood and buccal epithelial cells. Blood originates from the lateral mesodermal plate, whereas buccal epithelial cells originate from ectoderm, along with skin. Mosaicism is unevenly distributed throughout the body, and a specific organ can be more commonly involved in mosaicism. Furthermore, the distribution could be different according to each genotypes. Several hypotheses have been raised to explain this phenomenon including lineage-specific selection and oncogene-induced senescence [3].

There was no case of any malignancy among our patients. Contrary to *PTEN-*hamartoma tumor syndrome, which is caused by variants in the negative regulator of the PI3K pathway, only a few malignancies have been reported in patients with PROS [1, 3, 9]. In patients with MCAP, two with meningioma, one with leukemia, one with subcutaneous lipoma and two with Wilms’ tumor have been reported [21]. This might be because mutational hotspots for PROS and cancer were distinguished and the tumorigenic activity of MCAP-related variants seemed to be less than that of oncogenic variants. However, larger cohorts and longer follow-up duration are needed to determine whether the cancer risk is increased in patients with PROS.

To date, the treatment of patients with PROS has been based mainly on addressing symptoms when the overgrowth causes organ dysfunction, and operations such as foramen magnum decompression or VP shunt are required when the hydrocephalus becomes aggravated. Inhibitors of the PI3K/AKT/mTOR signaling pathway, originally developed as anticancer agents, are now showing encouraging outcomes in preclinical models and off-label trials [5, 21, 32, 33]. In particular, a phase I trial of alpelisib, a specific PI3K inhibitor, has started on the strength of successfully treated cases [5, 6]. Therefore, early diagnosis and precise molecular confirmation of PROS are emphasized increasingly. Eight of our patients (67%) had undergone various genetic evaluations before the diagnosis of MCAP. To make a prompt and accurate diagnosis, we should specify the clinical indications needed to investigate somatic mosaicisms and suggest the use of high-depth NGS panel sequencing of affected tissues ahead of WES using peripheral blood. In addition, obtaining adequate tissue samples from affected lesions tends to produce a higher variant detection rate.

In patients with MCAP, overgrowth except for macrocephaly is usually not severe enough to be detected by prenatal screening, and only one case in our study showed abnormal prenatal ultrasonography findings. Also in cases of non-MCAP PROS, overgrowth is often aggravated with age and only extremely severe cases might be suspected prenatally. Thus, further studies are needed to establish guidelines for the prenatal diagnosis of postzygotic mosaic conditions including PROS.

## Conclusions

We present 12 patients who satisfied the strict clinical diagnostic criteria of MCAP and were diagnosed by the molecular identification of mosaic *PIK3CA* variants. Using high-depth NGS panel sequencing, allele frequencies of mosaicisms even lower than 10% were detected successfully. To administer target therapy in young patients as effectively as possible and manage the patients better, establishing clinical indications and strategies for molecular diagnosis and improving the diagnostic yield will be needed.

## Data Availability

The datasets used and/or analysed during the current study are available from the corresponding author on reasonable request.

## Abbreviations

AKT: Protein kinase B
CLOVES: Congenital lipomatous overgrowth, vascular malformations, epidermal nevi, scoliosis/skeletal and spinal syndrome
FAO: Fibroadipose hyperplasia or overgrowth
HHML: Hemihyperplasia multiple lipomatosis
MCAP: Megalencephaly-capillary malformation-polymicrogyria syndrome
MRI: Magnetic resonance imaging
mTOR: Mammalian target of the rapamycin
NGS: Next-generation sequencing
PI3K: Phosphatidylinositol 3-kinase
PROS: PIK3CA-related overgrowth spectrum
VAF: Variant allele frequency
VP: Ventriculoperitoneal
WES: Whole-exome sequencing
